# Role of *Toxoplasma gondii* calcium-binding protein (*Tg*pCaBP) and its implications for the development of attenuated live vaccines

**DOI:** 10.1128/spectrum.01827-26

**Published:** 2026-09-17

**Authors:** Yiwei Zhang, Hongyu Li, Yuqi Jing, Weisong Sun, Bin Wei, Zhiming Wei, Ning Jiang

**Affiliations:** 1 Key Laboratory of Livestock Infectious Diseases, Ministry of Education, and Key Laboratory of Ruminant Infectious Disease Prevention and Control (East), Ministry of Agriculture and Rural Affairs, College of Animal Science and Veterinary Medicine, Shenyang Agricultural University 98428, Shenyang, China; University of Illinois Urbana-Champaign, Urbana, Illinois, USA

**Keywords:** *Toxoplasma gondii*, calcium-binding protein, attenuated live vaccines, effector memory T cell

## Abstract

*Toxoplasma gondii *is an obligate intracellular protozoan parasite and the causative agent of toxoplasmosis in humans and animals. To date, an ideal vaccine against toxoplasmosis has not yet been developed. Our previous study demonstrated that deletion of the *Tg*p*CaBP* gene, which encodes a calcium-binding EF-hand protein, impaired calcium flux, invasion, and egress, thereby reducing *T. gondii* virulence. However, the mechanisms underlying this loss of virulence and the potential of RHΔ*Tg*p*CaBP* as a live attenuated vaccine remain unclear. Here, we showed that *Tg*p*CaBP* deletion significantly downregulated the expression of proteins associated with invasion, egress, and pyrimidine metabolism. *In vivo* evaluations revealed that co-administration of RHΔ*Tg*pCaBP and sulfadiazine showed limited pathological adverse effects in mice—all mice survived immunization with 100 tachyzoites without obvious organ injury, and conferred protection against lethal wild-type RH strain challenge (≤1,000 tachyzoites). Flowcytometry assays demonstrated that RHΔ*Tg*pCaBP strain immunization increased IFN-γ production in circulating CD4^+^ and CD8^+^ T cells and the frequencies of splenic CD4^+^ and CD8^+^ effector memory T cells, memory B cells, and activated B cells. In conclusion, *Tg*p*CaBP* plays an important role in regulating the virulence of *T. gondii* by modulating the expression of proteins related to pyrimidine metabolism, invasion, and egress. Co-immunization with 100 RHΔ*Tg*pCaBP tachyzoites and sulfadiazine shows favorable safety profiles and protective efficacy in mice, triggers observable immune memory, and may serve as a promising candidate for an attenuated live vaccine against toxoplasmosis.

**IMPORTANCE:**

Toxoplasmosis, caused by *T. gondii*, poses substantial public health and economic burdens worldwide. Although medicinal plants are traditionally the most promising source for the treatment of toxoplasmosis, no effective therapeutic regimen has been developed to date. Thus, the development of safe, effective, and long-lasting vaccines has become key for the prevention and control of toxoplasmosis. This study demonstrated that the deletion of *TgpCaBP*, a gene encoding an EF-hand calcium-binding protein, profoundly attenuated the virulence of the virulent RH strain by disrupting key pathogenic processes. More importantly, the RHΔ*TgpCaBP* strain, administered in combination with sulfadiazine, emerged as a promising live attenuated vaccine candidate. It exhibited favorable safety profiles in mice and elicited an immunomodulatory trend. Although further optimization is warranted, this study establishes a foundational framework for developing live attenuated vaccines against toxoplasmosis.

## INTRODUCTION

*Toxoplasma gondii* is a significant obligate intracellular protozoan parasite (1, 2) that can infect all warm-blooded vertebrates, including humans, and is primarily transmitted through the ingestion of tissue cysts in undercooked or raw meat or through contaminated food and water (3). The impact of *T. gondii* on public health is exacerbated by its ability to cause severe disease in immunocompromised individuals and in developing fetuses (3). Moreover, *T. gondii* poses a significant threat to animal husbandry, as this parasite is prevalent in various animal species, including wild canids and carnivores, where it can cause clinical and subclinical infections (4). The economic impact of *T. gondii* on animal husbandry is notable, as infections can lead to reproductive failure, such as abortion in livestock, thereby affecting productivity and economic returns (5). Therefore, effective prevention and control strategies against toxoplasmosis are urgently needed to protect public health and animal welfare.

Currently, the major challenge in the treatment of toxoplasmosis lies in eliminating tissue cysts during the chronic infection stage. The traditional first-line drug combination of pyrimethamine and sulfadiazine can inhibit folate metabolism in the parasites but is associated with side effects such as myelosuppression and fails to penetrate the cyst wall to kill bradyzoites (6). Although medicinal plants are traditionally the most promising source for the treatment of toxoplasmosis, no effective therapeutic regimen has been developed to date (7). Thus, the development of safe, effective, and long-lasting vaccines has become key for the prevention and control of toxoplasmosis. Over the past few decades, safer vaccine approaches, including but not limited to, recombinant immunogenic protein vaccines, naked DNA vaccines, viral/bacterial recombinant vector vaccines, and nanoparticles, have continuously been explored (8–11). Despite the promising potential of these strategies, attenuated live vaccines derived from *T. gondii* strains have been demonstrated to possess more robust protective efficacy than vaccines based on a single or a few antigens do (12–15). To date, multiple efforts have been made to generate attenuated vaccine candidates using gene depletion strategies, such as RHΔompdcΔuprt (16), ME49Δcdpk2 (17), and ME49 Δldh (18), some of which have achieved favorable immunoprotective effects. However, there is only one commercially available vaccine, Toxovax, based on the tachyzoites of the *T. gondii* S48 strain, which has several limitations (19).

Calcium (Ca²^+^) acts as a pivotal second messenger that governs nearly all vital biological processes in *T. gondii*, including gliding motility and host cell invasion, which are collectively essential for parasite survival (20, 21). Calcium-binding proteins (CaBPs) serve as key regulators that maintain intracellular Ca²^+^ homeostasis and mediate Ca²^+^ signal transduction (22). Our previous study identified and characterized a novel EF-hand containing CaBP in *T. gondii*, designated *Tg*pCaBP. Deletion of the *Tg*p*CaBP* gene resulted in severe defects in Ca²^+^ storage and efflux and led to impaired gliding motility, host cell invasion, intracellular growth, microneme secretion, and parasite egress (23). Consequently, the virulence of RHΔ*Tg*pCaBP parasites was significantly attenuated in a mouse model (23). Whether the RHΔ*Tg*pCaBP strain can be further optimized and developed into a safe and effective live attenuated vaccine remains unknown.

Here, we performed a quantitative proteomic analysis to elucidate the molecular changes induced by *Tg*p*CaBP* deletion. We found that the loss of *Tg*p*CaBP* significantly downregulated proteins associated with parasite invasion, egress, and pyrimidine metabolism. On the basis of these findings, we further inhibited the pyrimidine metabolic pathway of RHΔ*Tg*p*CaBP* with sulfadiazine. Our results demonstrated that co-immunization with 100 RHΔ*Tg*pCaBP tachyzoites and sulfadiazine was safe in mice and provided complete protection against lethal challenge with less than 1,000 tachyzoites of the wild-type (WT) RH strain. Mechanistically, vaccination with RHΔ*Tg*CaBP notably increased the frequencies of splenic CD4^+^ effector memory T cells, CD8^+^ effector memory T cells, memory B cells, and activated B cells. Furthermore, RHΔTgCaBP immunization significantly upregulated the IFN-γ secretion and downregulated expression of key immune checkpoint molecules, including PD-1 and CTLA-4, in circulating CD4^+^ and CD8^+^ T cells. Overall, our study provides a promising candidate for the development of live attenuated vaccines against toxoplasmosis.

## RESULTS

### *Tg*p*CaBP* knockout downregulated DNA replication- and pyrimidine metabolism-related proteins but did not affect the intracellular proliferation of *Toxoplasma gondii*

**Fig 1:**
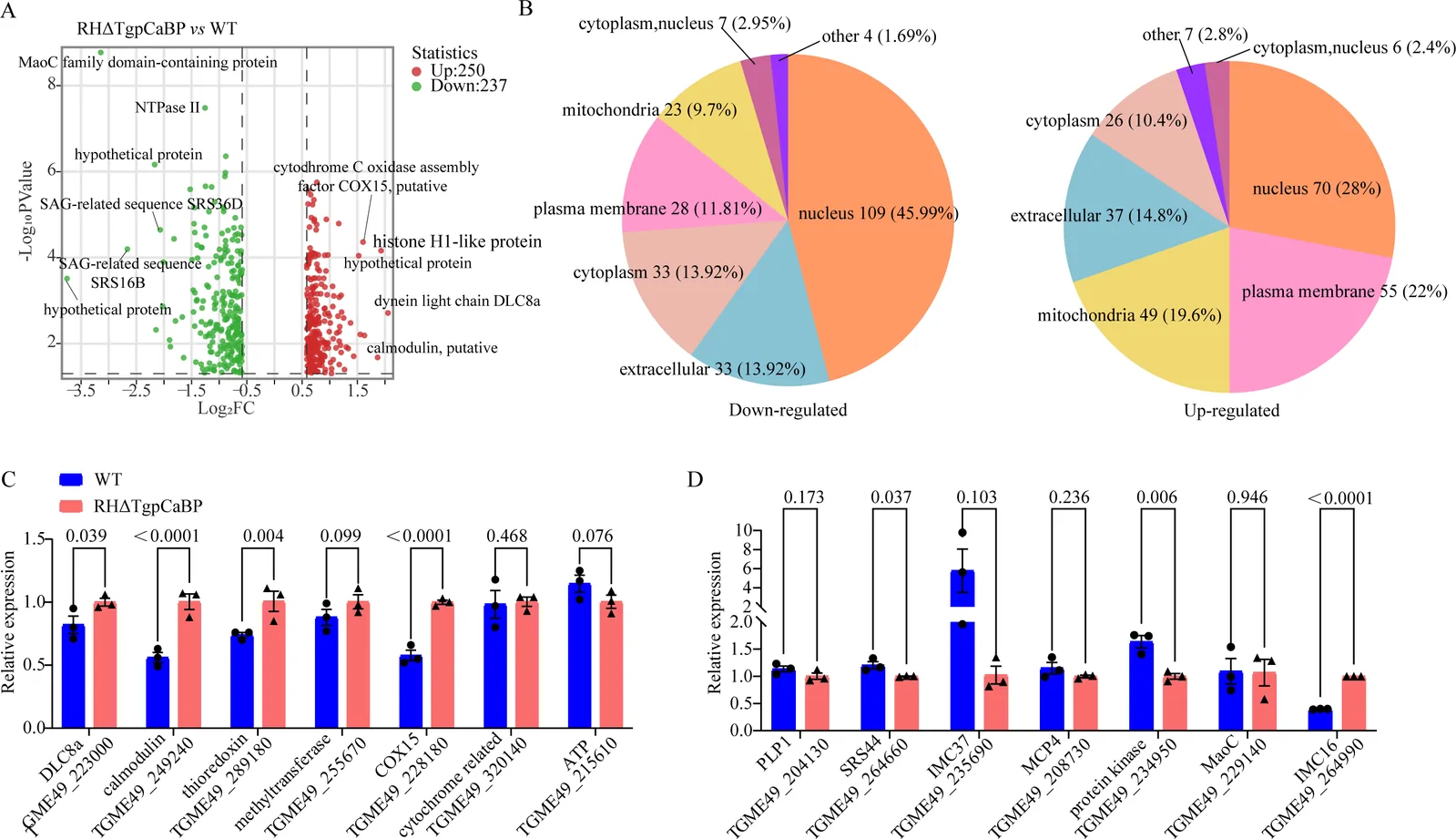
Proteomic analysis and Q-PCR validation in *Tg*p*CaBP*-knockout parasites. (**A**) Volcano plot showing differentially expressed proteins in the RHΔ*Tg*pCaBP group vs the WT group. Numbers of genes with log2 fold change values of >0.5 or <−0.5 and an adjusted *P*-value of <0.05 are shown. Red dots represent proteins upregulated in the RHΔ*Tg*pCaBP group, while green dots represent proteins downregulated in this group. (**B**) Subcellular localization of downregulated (left panel) and upregulated (right panel) proteins in the RHΔ*Tg*pCaBP group. (**C**) Q‒PCR validation of a subset of proteins whose expression was upregulated in the RHΔ*Tg*pCaBP group. (**D**) Q‒PCR validation of a subset of proteins whose expression was downregulated in the RHΔ*Tg*pCaBP group; β-tubulin was used as the reference gene (*n*  =  3; unpaired two-tailed Student’s *t*-test; the data are presented as the means  ±  SEMs). The specific primers used for panels C and D are listed in Table S1.

To elucidate the molecular mechanisms underlying the reduced virulence of the RHΔ*Tg*p*CaBP* strain, proteomic analysis was performed. Quality control analysis of the proteomic data confirmed the reliability of the detection results (Fig. S1), and 250 upregulated and 237 downregulated proteins were found after *TgpCaBP* was knocked out (Fig. 1A). Subcellular localization analysis revealed that more than 45% of the downregulated proteins were localized in the nucleus upon *Tg*p*CaBP* knockout, whereas only 28% of the upregulated proteins were localized in the nucleus (Fig. 1B). Studies have demonstrated that intracellular Ca²^+^ modulates diverse cellular processes, including cell proliferation and protein secretion, in both eukaryotes and prokaryotes, with calcium-binding proteins (CaBPs) serving as central regulators of these events (24). These findings suggest that *Tg*p*CaBP* deletion may impair nuclear functions related to DNA replication and gene expression, which likely contributes to the attenuated virulence of *T. gondii*. To verify the accuracy of the proteomic results, several key differentially expressed proteins (DEPs) were selected for Q-PCR validation, and the results revealed that the transcriptional levels of these proteins were mostly consistent with their proteomic expression patterns (Fig. 1C and D).

**Fig 2:**
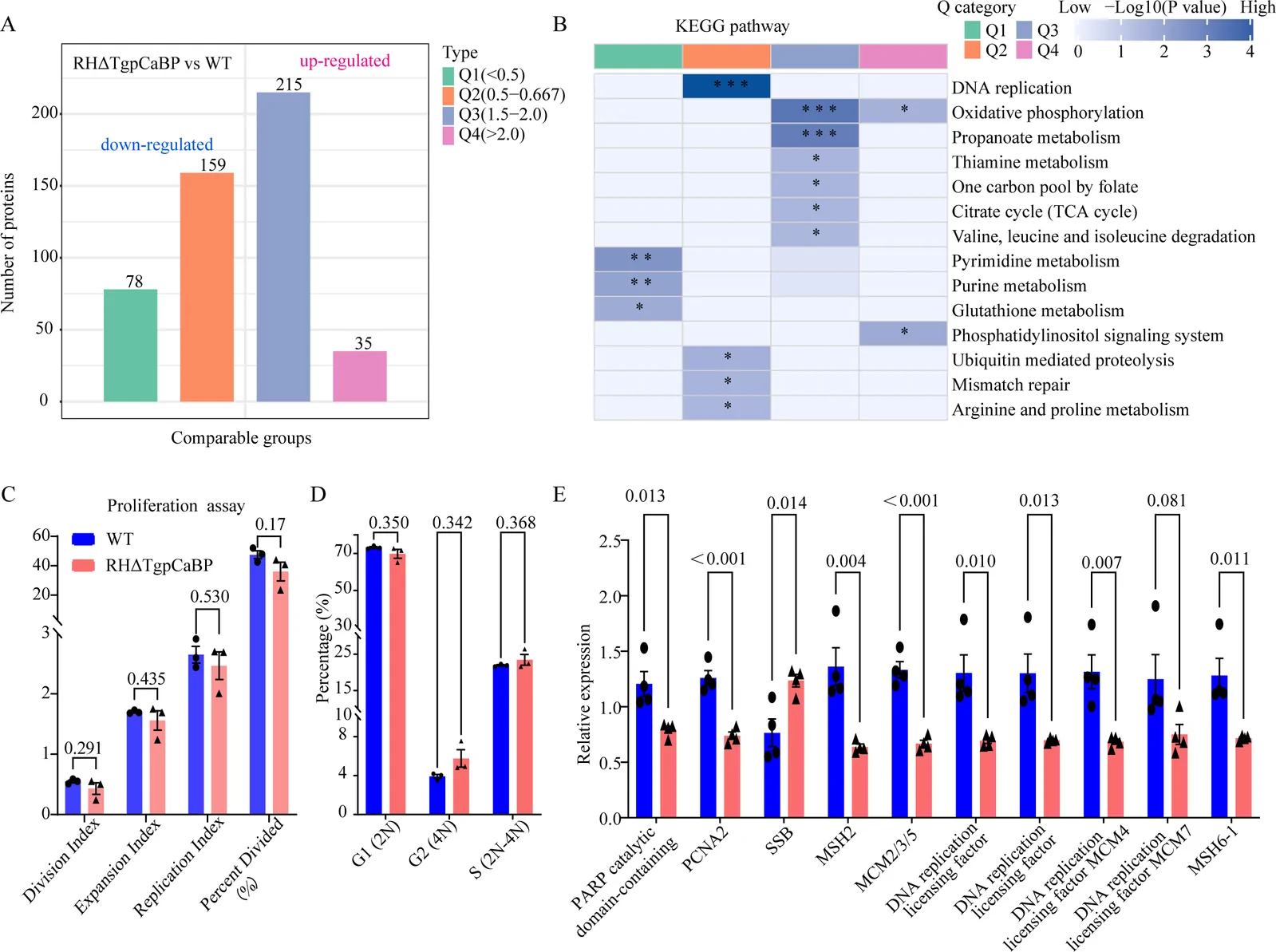
Functional enrichment analysis of the differentially expressed proteins and assessment of RHΔ*Tg*pCaBP and WT parasite proliferation. (**A**) Categorization of the differentially expressed proteins. Differentially expressed proteins were classified into four distinct functional groups based on their fold-change values: Q1 (<0.5, downregulated), Q2 (0.5–0.667, downregulated), Q3 (1.5–2.0, upregulated), and Q4 (>2.0, upregulated). The bar chart displays the number of proteins within each category. (**B**) KEGG analysis of the four protein categories. A heatmap was generated using hierarchical clustering based on *P* values from Fisher’s exact test to visualize the enrichment of functional pathways across the four Q groups in Fig. 2A. The color intensity of each cell reflects the magnitude of enrichment significance, expressed as −log_10_(*P* value), with darker shades indicating greater statistical significance. Significance levels are denoted as follows: *P < 0.05, **P < 0.01, and ***P < 0.001. (**C**) The proliferation of RHΔTgpCaBP parasites compared with that of the WT parasites was evaluated by flow cytometry (*n*  =  3; unpaired two-tailed Student’s *t*-test; the data are presented as the means  ±  SEMs). (**D**) The proliferation cycle of the RHΔTgpCaBP strain compared with that of the WT parasites was analyzed by flow cytometry (*n*  =  3; unpaired two-tailed Student’s t test; the data are presented as the means  ±  SEMs). (**E**) Expression levels of proteins associated with DNA replication were assessed (*n*  =  4; unpaired two-tailed Student’s *t*-test; the data are presented as the means  ±  SEMs).

Furthermore, the DEPs were classified into four categories on the basis of their extent of differential expression (Fig. 2A). KEGG functional enrichment analysis was subsequently performed to determine the biological functions of the DEPs. Notably, the downregulated DEPs exhibited enrichment mainly in biological processes related to pyrimidine metabolism (Q1) and DNA replication (Q2) (Fig. 2B). Flow cytometry was used to evaluate the intracellular proliferation and cell cycle progression of the RHΔ*Tg*pCaBP parasites. Notably, no significant differences in the intracellular proliferation capacity (Fig. 2C) or cell cycle distribution (Fig. 2D) were detected between the RHΔ*Tg*pCaBP strain and the wild-type (WT) strain after 24 h of *in vitro* culture, which is highly consistent with our previously published study (23). In-depth analysis of the DEPs related to DNA replication revealed that most of the downregulated proteins were classified as “DNA replication silencing factors”, which might explain why the intracellular proliferation of the RHΔ*Tg*pCaBP parasites was not affected (Fig. 2).

### *TgpCaBP* knockout downregulated the expression of proteins related to invasion, motility, and egress

**Fig 3:**
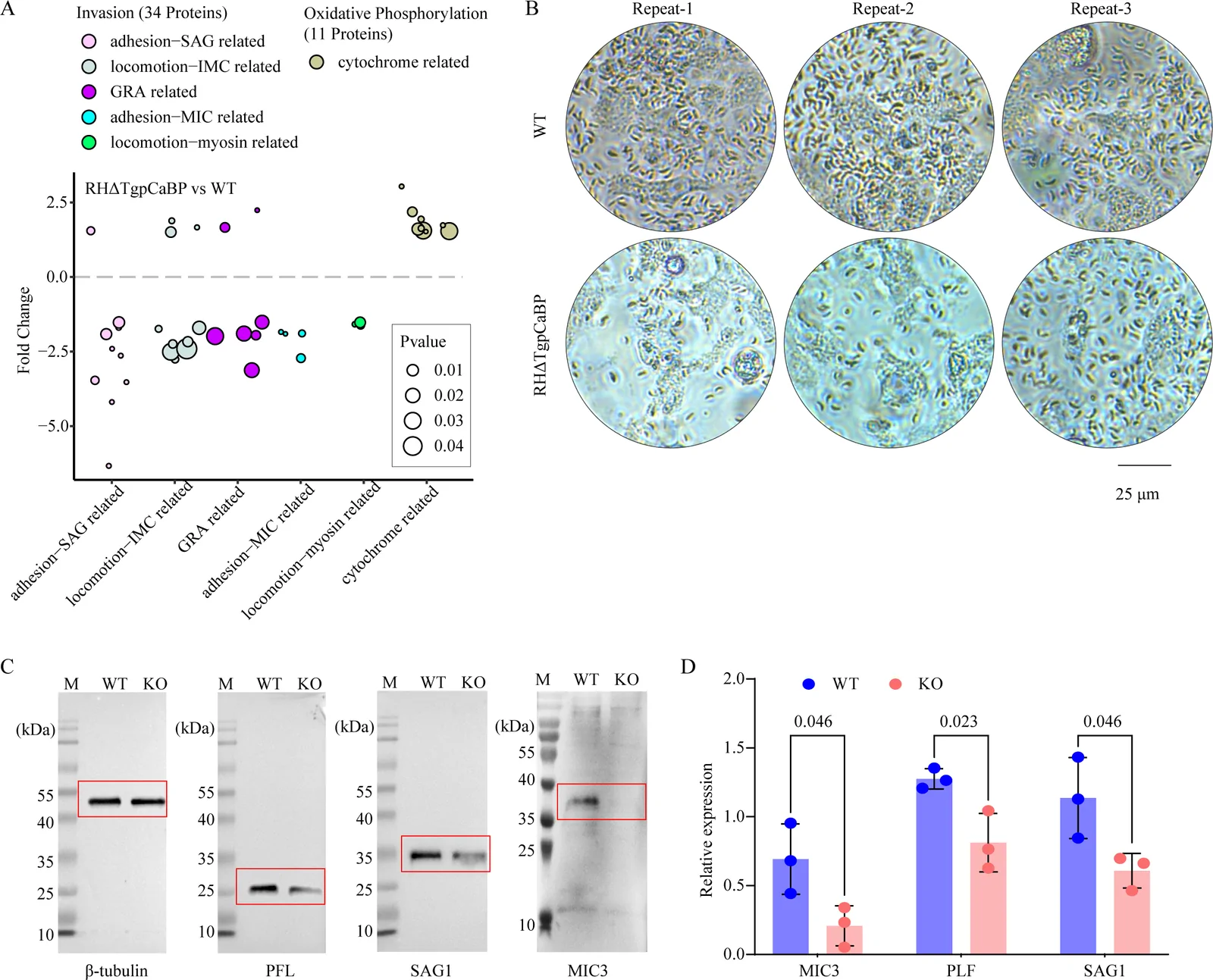
The expression of invasion- and egress-related proteins is downregulated upon knockout of the *Tg*p*CaBP* gene. (**A**) Bubble map of the results of the proteomic analysis showing that the expression levels of DEPs related to invasion and locomotion in the RHΔTgpCaBP strain were downregulated compared with those in the WT strain. Genes with log2 fold change values of >0.5 or <−0.5 and an adjusted *P*-value of <0.05 are shown. Detailed information is provided in Table S3. (**B**) Microscopy images of RHΔTgpCaBP and WT parasites after 24 h of *in vitro* culture. Scale bar = 25 µm. A significant reduction in parasite number was observed in the RHΔTgpCaBP group. (**C**) Western blot analysis of the protein expression levels of PFL, SAG1, and MIC3. M: Protein molecular weight marker (kDa). WT: wild-type parasites; KO: RHΔTgpCaBP parasites. Red boxes highlight the target protein bands. β-Tubulin served as the reference protein. (**D**) Quantitative grayscale analysis of the Western blots. The relative expression levels of MIC3, PFL, and SAG1 in the WT (blue bars) and RHΔTgpCaBP (red bars) groups were normalized to those of β-tubulin with ImageJ (*n*  =  3; unpaired two-tailed Student’s *t*-test, with the data denoted as the means  ±  SEMs).

Furthermore, the top 100 differentially DEPs associated with parasite invasion and motility, which are critical for *T. gondii* pathogenesis, were selected for further analysis (Fig. S2). The results revealed that proteins involved in parasite invasion and motility were significantly downregulated in RHΔ*Tg*pCaBP compared with their expression levels in WT parasites (Fig. 3A). Consistent with these proteomic findings, an *in vitro* proliferation assay revealed that the number of RHΔ*Tg*pCaBP tachyzoites was significantly reduced after several generations of proliferation (24 h) compared with that of the WT parasites (Fig. 3B). Since our previous results demonstrated that *Tg*pCaBP knockout did not affect the intracellular proliferation capacity of parasites (Fig. 2C through E), we hypothesized that the reduced number of parasites might be due to impaired invasion and escape capabilities. To validate this hypothesis, Western blot (WB) analysis was performed to determine the expression levels of key egress- and invasion-related proteins, such as perforin-like protein (PFL; TGME49_204130), surface antigen 1 (SAG1; TGVEG_233460), and microneme protein 3 (MIC3; TGME49_319560). The results confirmed that the expression of these proteins was significantly downregulated after *Tg*pCaBP knockout (Fig. 3C and D). A previous study showed that PFLs are essential for *T. gondii* to invade host organisms and complete their life cycle (25). SAG1 has been confirmed to be a crucial ligand of *T. gondii* and plays a pivotal role in the tachyzoite invasion of host cells by enabling tachyzoite–host receptor binding (26). Furthermore, MIC3 has a high affinity for host cells, with its CBL domain being essential for *T. gondii*–cell adhesion (27). These data reveal a correlative link between *Tg*pCaBP deletion and downregulated expression of invasion- and egress-associated proteins, consistent with our previously published functional characterization study that *Tg*pCaBP is required to sustain normal invasion, egress, and *in vitro* proliferative capacity of *T. gondii* (23).

### Co-immunization with RHΔ*Tg*pCaBP strain parasites plus sulfadiazine achieves favorable safety and protective effects in mice

Our previous proteomic analysis revealed that many proteins that were significantly downregulated in RHΔ*Tg*pCaBP parasites are involved in the pyrimidine metabolism pathway (Fig. 2B). Pyrimidine metabolism is a critical component of nucleotide metabolism, which is essential for the survival and pathogenicity of *T. gondii* (28, 29). Notably, nucleotide metabolism in protozoan parasites is being increasingly explored as a drug target (30). Moreover, nucleoside antimetabolites have been suggested to be effective drug candidates against various protozoan parasites (31–33). To further increase the safety of our potential live attenuated vaccine, we sought to further weaken the pyrimidine metabolism pathway in RHΔ*Tg*pCaBP parasites by treating them with sulfadiazine, which inhibits folate synthesis in parasites, thereby affecting the metabolism of pyrimidine nucleotides (34, 35).

**Fig 4:**
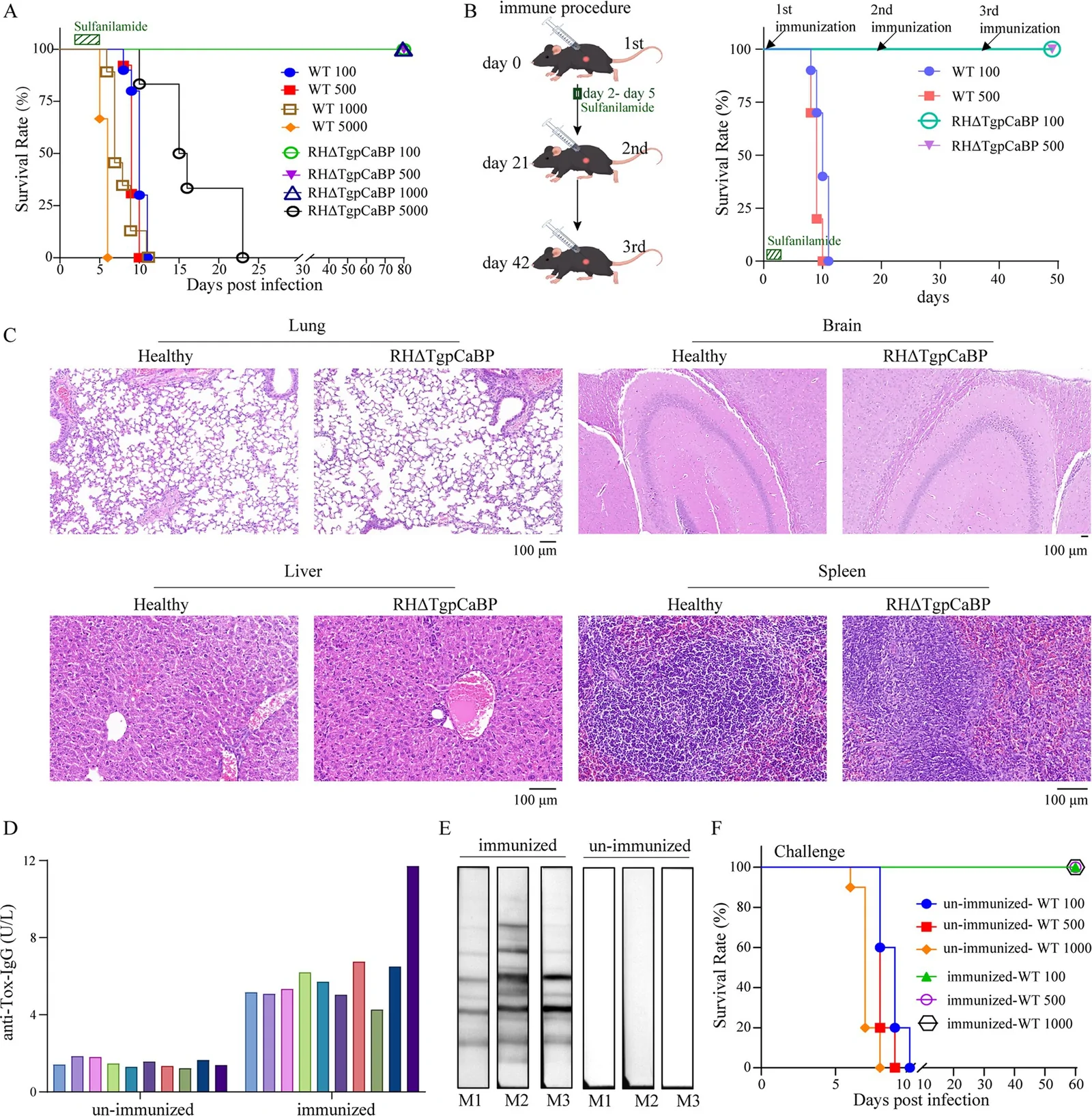
Evaluation of the virulence of RHΔ*Tg*CaBP and its potential as an attenuated live vaccine. (**A**) The survival rate of mice infected with the RHΔ*Tg*CaBP or WT (100, 500, 1000, and 5000 tachyzoites) was monitored over time. At 48 h post-infection, all mice were provided drinking water containing sulfadiazine at a concentration of 400 mg/L for 3 days, after which the water was replaced with regular drinking water. (**B**) Schematic diagram of the immunization procedure (left panel, three-dose immunization) and survival statistics of the immunized mice (right panel). The mice were immunized on Days 0, 21, and 42. The survival rates of the mice immunized with RHΔ*Tg*CaBP and WT were recorded. Schematic diagram constructed with Figdraw (Authorization ID: UTAWA44495). (**C**) Pathological changes in the lungs, brain, liver, and spleen of mice after immunization. Tissue sections from healthy mice (control) and RHΔ*Tg*CaBP-immunized mice (100 tachyzoites) were stained with HE (scale bars = 100 µm). (**D**) ELISA detection of IgG levels against total RH protein in serum samples from unimmunized and immunized mice. (**E**) Western blot detection of IgG levels against total RH protein in serum samples from unimmunized and immunized mice. Serum samples were diluted 1:1,000. Lanes M1–M3 represent three different mice in each the unimmunized and immunized groups. (**F**) Unimmunized and immunized mice were challenged with parasites, and the survival rates were monitored over time to evaluate the protective effect of RHΔ*Tg*CaBP immunization.

To initially evaluate the virulence of the RHΔ*Tg*pCaBP parasites, mice were infected with different doses (100, 500, 1,000, and 5,000 tachyzoites) of the RHΔ*Tg*pCaBP or WT RH strain, and the survival rates of the mice were monitored over time (Fig. 4A). At 48 h post-infection, all groups of mice were provided drinking water containing 400 mg/L sulfadiazine for three consecutive days, followed by access to regular drinking water. The results revealed that mice infected with RHΔ*Tg*pCaBP less than 1,000 tachyzoites further treated with sulfadiazine achieved 100% survival (Fig. 4A). In contrast, even upon cotreatment with sulfadiazine, mice infected with the WT *T. gondii* RH strain died 6–11 days post-infection (Fig. 4A).

To systematically evaluate the safety and protective efficacy of the RHΔ*Tg*pCaBP strain as an attenuated live vaccine, four experimental groups of C57BL/6 female mice were established, and immunization was performed as illustrated in Fig. 4B (left panel), with three doses administered on Days 0, 21, and 42. In accordance with the virulence evaluation protocol, 48 h after the first immunization, all the mice in each group were administered 400 mg/L sulfadiazine in the drinking water (the water bottles were wrapped in tinfoil for light protection) for three consecutive days, after which the water was replaced with regular drinking water; sulfadiazine was not added to the drinking water during the subsequent two immunization procedures. Survival analysis revealed that mice immunized with 100 or 500 RHΔ*Tg*pCaBP tachyzoites achieved 100% survival (Fig. 4B, right panel). Seven days after the third immunization procedure, the mice were euthanized, and the liver, lung, brain, and spleen were removed for safety evaluation. Pathological changes in these organs were observed by hematoxylin‒eosin (HE) staining. The pathological results revealed no significant differences in liver, lung, brain, or spleen tissues between the healthy group and the group immunized with 100 RHΔ*Tg*pCaBP tachyzoites (Fig. 4C). However, several mice immunized with 500 RHΔ*Tg*pCaBP tachyzoites exhibited mild neurological symptoms, and partial structural damage was observed in the brain tissue (Fig. S3). On the basis of these findings, 100 RHΔ*Tg*pCaBP tachyzoites were selected as the optimal immunization dose for subsequent experiments.

The protective efficacy of the RHΔ*Tg*pCaBP strain was evaluated by detecting serum IgG levels against total RH proteins after the third immunization and challenge with WT RH tachyzoites. An enzyme-linked immunosorbent assay (ELISA) was performed to detect IgG levels against total RH protein in serum samples from unimmunized and immunized mice, and the results revealed a significant increase in serum IgG levels in immunized mice compared with those in unimmunized mice (Fig. 4D), which was verified by Western blot (WB) analysis (Fig. 4E). Subsequently, the mice in the unimmunized and immunized groups were intraperitoneally challenged with WT RH tachyzoites, and survival analysis revealed that all the mice in the immunized group survived without obvious clinical symptoms, whereas all the unimmunized (negative control) mice died of acute infection within 10 days (Fig. 4F). Taken together, these results demonstrate that immunization with the RHΔ*Tg*pCaBP strain parasites (co-treated with 400 mg/L sulfadiazine for three consecutive days at the first immunization procedure) can provide 100% protection against challenge with low-dose virulent WT RH tachyzoites (≤1,000 tachyzoites).

### RHΔ*Tg*pCaBP immunization increases interferon-γ (IFN-γ) expression in the circulating T cells of mice after parasite challenge

**Fig 5:**
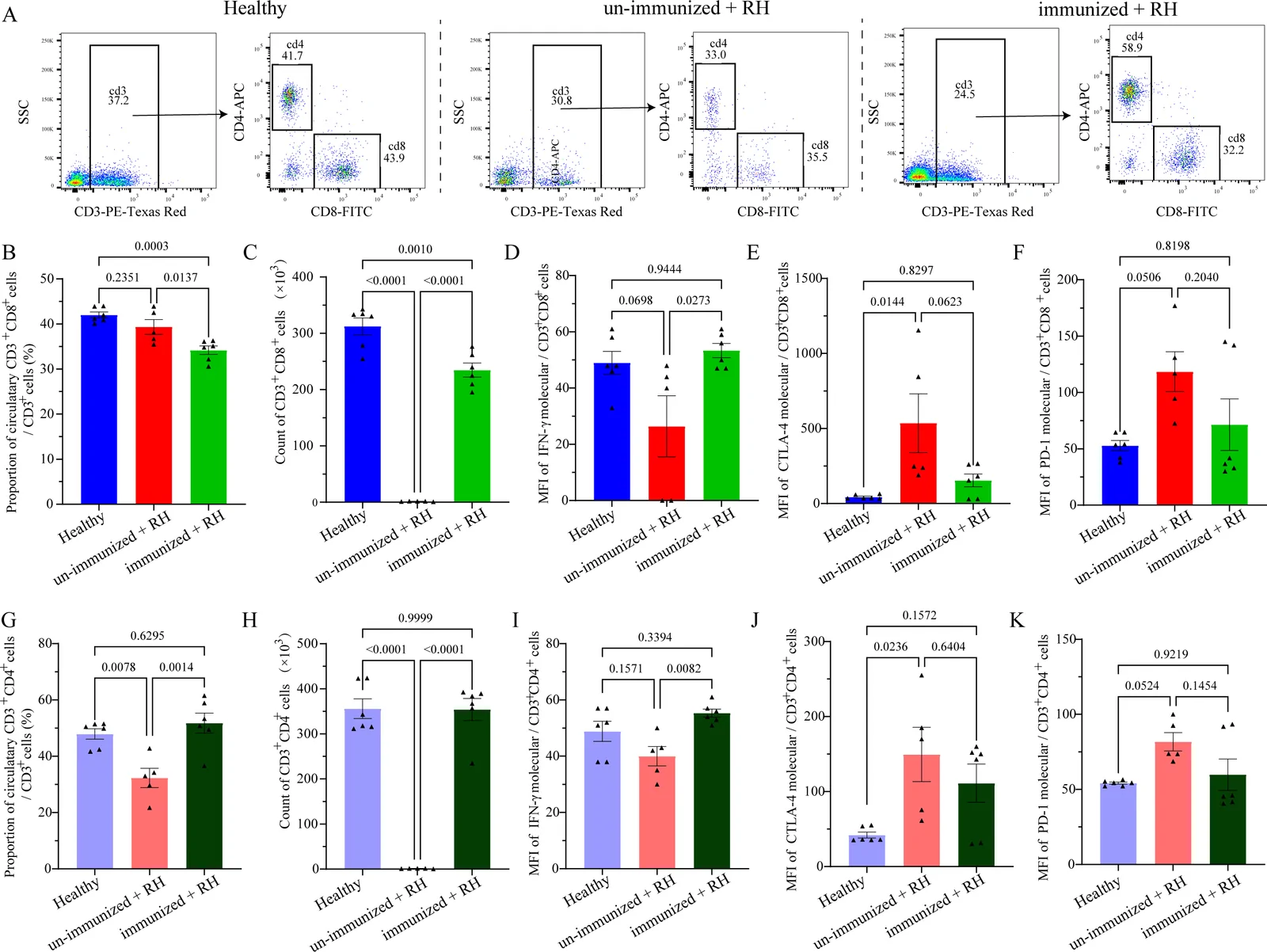
Changes in the expression of IFN-γ, CTLA-4, and PD-1 on circulating T cells in mice after immunization with RHΔ*Tg*CaBP and subsequent parasite challenge. (**A**) Flow cytometry gating strategy to analyze T-cell subsets. (**B–F**) Statistical analysis of the proportion of CD3^+^CD8^+^ cells (**B**), the number of CD3^+^CD8^+^ cells in 100 μL of blood (**C**), and the expression of IFN-γ (**D**), CTLA-4 (**E**), and PD-1 (**F**) in CD3^+^CD8^+^ cells. (**G–K**) Statistical analysis of the proportion of CD3^+^CD4^+^ cells (**B**), the number of CD3^+^CD4^+^ cells in 100 μL of blood (**C**), and the expression of IFN-γ (**D**), CTLA-4 (**E**), and PD-1 (**F**) in CD3^+^CD4^+^ cells (*n* = 5–7; unpaired two-tailed Student’s *t*-test, with data denoted as the means ± SEMs).

To characterize the effector functions of T cells, we analyzed the proportions and numbers of circulating T cells, as well as the expression of IFN-γ, cytotoxic T-lymphocyte associated protein-4 (CTLA-4), and programmed death receptor 1 (PD-1) in T cells. With respect to CD8^+^ T cells, the proportion of circulating CD3^+^CD8^+^ T cells was significantly lower in the immunized +RH group than in the healthy and unimmunized + RH groups (Fig. 5A and B). However, the absolute count of CD3^+^CD8^+^ T cells sharply decreased in the unimmunized + RH group compared with that in the healthy and immunized + RH groups (Fig. 5C). Functional analysis revealed that the expression of IFN-γ in CD3^+^CD8^+^ T cells was markedly greater in the RHΔ*Tg*pCaBP immunized group than in the unimmunized + RH group (Fig. 5D). Moreover, the expression levels of the immune checkpoint molecules CTLA-4 and PD-1 on CD3^+^CD8^+^ T cells were elevated in the unimmunized + RH group compared with those in the healthy control group (although the difference was significant), while the expression levels of these factors were effectively reduced upon RHΔTgpCaBP immunization (Fig. 5E and F). With respect to CD4^+^ T cells, the proportion and absolute number of circulating CD3^+^CD4^+^ T cells were significantly greater in the immunized + RH group than in the unimmunized + RH group (Fig. 5G and H). The expression of IFN-γ in CD3^+^CD4^+^ T cells was significantly greater in the immunized + RH group than in the unimmunized + RH group (Fig. 5I). Similar to the results with CD8^+^ T cells, CTLA-4 and PD-1 expression on CD3^+^CD4^+^ T cells was upregulated in the unimmunized + RH group, but RHΔTgpCaBP immunization reduced their expression, although not significantly (Fig. 5J and K). Collectively, these results demonstrate that RHΔTgpCaBP immunization enhanced the effector function of both CD3^+^CD4^+^ and CD3^+^CD8^+^ T cells, as evidenced by increased IFN-γ production and downregulated expression of inhibitory immune checkpoint molecules.

### RHΔ*Tg*pCaBP immunization induces significant increases in splenic memory T cells and B cells in mice after parasite challenge

**Fig 6:**
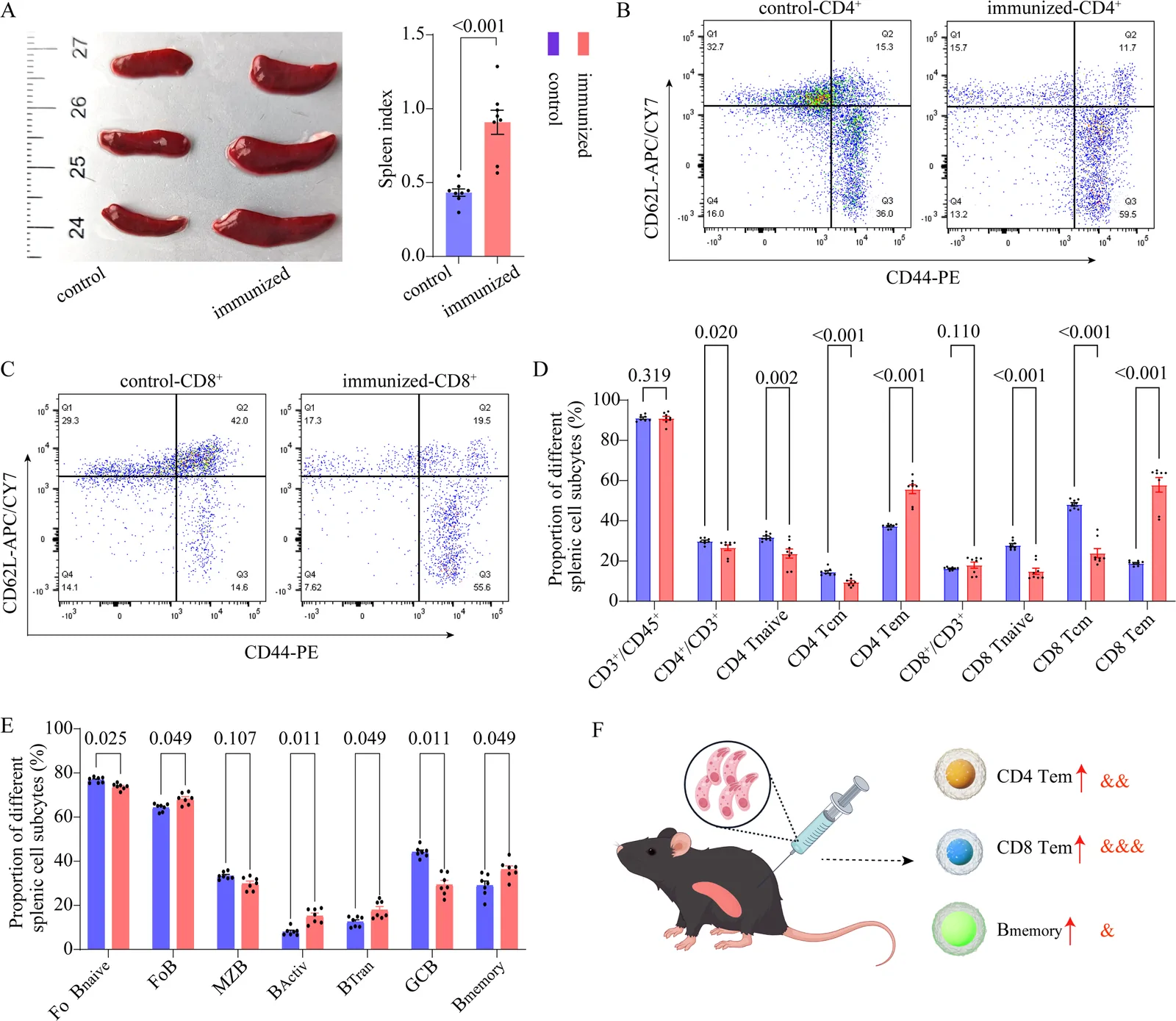
Changes in splenic memory T cells and B cells in mice after immunization with RHΔ*Tg*CaBP and subsequent parasite challenge. (**A**) At 30 days post-challenge, mice were sacrificed. The spleens were observed (left panel), and the spleen index (right panel) was calculated as follows: spleen index = spleen wt (mg) × 10/body weight (G) × 100%. (**B**) Gating strategy for flow cytometry analysis of CD4^+^ memory T cells. (**C**) Gating strategy for flow cytometry analysis of CD8^+^ memory T cells. (**D**) Statistical analysis of CD4^+^ and CD8^+^ memory T cells: compared with those in the healthy control group, the proportions of splenic CD4^+^ T_em_s and CD8^+^ T_em_s in immunized mice were significantly greater. (**E**) Statistical analysis of memory B cells: compared with those in the healthy control group, the proportions of B_memory_s and B_Active_s in the spleens of immunized mice were significantly greater. (**F**) Schematic representation of enhanced immune memory upon immunization. The symbols “&,” “&&,” and “&&&” indicate the extent of relative increase in each immune cell subset, where the number of symbols corresponds to the magnitude of upregulation. The schematic diagram was constructed with Figdraw (Authorization ID: UTAWA44495) (*n* = 7; unpaired two-tailed Student’s *t*-test; the data are presented as the means ± SEMs).

To explore the protective mechanism of the RHΔ*Tg*pCaBP strain as an attenuated live vaccine in depth, we evaluated the changes in splenic immune memory T cells and B cells in immunized mice 30 days after challenge with 100 WT RH tachyzoites. The spleen index in the immunized group was significantly greater than that in the healthy control group (Fig. 6A), reflecting the regulatory effect of RHΔ*Tg*pCaBP immunization on splenic function. The flow cytometry results indicated that compared with those in the healthy control group, the proportions of splenic CD4^+^ effector memory T cells (CD4 T_em_) and CD8^+^ effector memory T cells (CD8 T_em_) in the immunized group were significantly greater (Fig. 6B through D). In addition, the proportion of splenic memory B cells was determined on the basis of the gating strategy shown in Fig. S4. Statistical analysis revealed that, compared with those in the healthy control group, the proportions of memory B cells (B_memory_) and active B cells (B_Active_) in the spleens of immunized mice were significantly greater (Fig. 6E). These results indicate that RHΔ*Tg*pCaBP immunization can effectively induce the generation of immune memory cells in the spleen.

## DISCUSSION

Toxoplasmosis, caused by the protozoan parasite *T. gondii*, poses a significant threat not only to human health but also causes substantial economic losses in the livestock industry, primarily due to inducing abortion in small ruminants and pigs, reducing the quality of animal products, and posing a considerable zoonotic risk (36–39). A range of drugs with antiparasitic properties are currently available for the treatment or mitigation of *T. gondii* infection. However, these therapeutic regimens are often limited in efficacy, have incomplete safety profiles, and may be associated with significant adverse effects (40–42). Thus, the development of a safe and effective vaccine against *Toxoplasma gondii* remains crucial for controlling toxoplasmosis (43).

In recent years, significant progress has been made in the design of candidate vaccines against *T. gondii*, encompassing a diverse array of platforms such as recombinant vaccines, DNA vaccines, subunit vaccines, live attenuated vaccines, and nanoparticle-based vaccines (43). While subunit and DNA vaccines have demonstrated partial immunoprotective efficacy, the prevailing consensus holds that live-attenuated *T. gondii* vaccines stand out among various vaccine platforms because they induce near-sterilizing immunity against infections caused by diverse *T. gondii* strains (18, 44–47). Here, we demonstrated that the deletion of *TgpCaBP*, a gene encoding an EF-hand calcium-binding protein, profoundly attenuated the virulence of the virulent RH strain by disrupting key pathogenic processes, including calcium-dependent invasion, egress, and pyrimidine metabolism (Fig. 1 to 3). More importantly, the RHΔ*Tg*pCaBP strain, whose pyrimidine pathway is further inhibited by sulfadiazine, emerged as a promising live attenuated vaccine candidate. It exhibited favorable safety profiles in mice and triggered detectable protective immunity and distinct cellular immune phenotypes. (Fig. 4 to 6).

The central role of calcium signaling in apicomplexan parasite biology is well established and involves essential processes, such as gliding motility and host cell egress (48, 49). Our proteomic data revealed that the absence of *Tg*p*CaBP* leads to the significant downregulation of proteins critical for *T. gondii* invasion and egress (Fig. 1 and 2). Notably, the expression of microneme protein 3 (MIC3) was almost completely suppressed in our study after the deletion of the *Tg*pCaBP gene (Fig. 3). These findings align with previous findings that an imbalance of calcium ions induces the deregulation of MIC secretion (50–52). Furthermore, our proteomic analysis revealed the significant downregulation of proteins associated with the pyrimidine metabolism pathway in the RHΔ*Tg*pCaBP strain. The pyrimidine metabolism pathway is indispensable for parasite proliferation (53), which suggests that *Tg*pCaBP may function as a broader regulatory nexus, linking calcium flux to metabolic reprogramming; however, this hypothesis warrants further investigation.

Since *T. gondii* is an intracellular parasite, a high concentration of specific IgG alone is insufficient to effectively control infection. Therefore, a cellular immune response is crucial for this protective effect (54, 55). We found that immunization with the RHΔ*Tg*pCaBP strain not only induced high levels of IgG but also upregulated the expression of IFN-γ (Fig. 5 and 6). Previous studies have shown that the administration of polyclonal rabbit anti-*T*. *gondii* IgG antibodies can prevent early mortality and pathology associated with tachyzoites in the brains of infected mice (56, 57). IFN-γ is the essential cytokine for controlling acute *T. gondii* infection, which functions primarily by activating macrophages to kill intracellular parasites and by inducing antiparasitic effector mechanisms in various host cells (58–60). PD-1 and CTLA-4 are suppressed immune checkpoints that inhibit the antimicrobial immune response of the host (61). Blockade of the PD-1-PD-L1 pathway restores CD8^+^ T-cell dysfunction, resulting in control of parasite reactivation and the prevention of mortality in chronically infected animals (62). Intriguingly, our study revealed that upon reinfection following immunization with the RHΔ*Tg*pCaBP strain, the expression of the inhibitory immune checkpoint molecules PD-1 and CTLA-4 was downregulated in circulating CD4^+^ and CD8^+^ T cells (Fig. 5). Previous research has shown that virus-like particles containing multiple antigenic proteins of *T. gondii* can induce memory T-cell and B-cell responses, which contribute to complete protection against *T. gondii* (ME49) challenge infection (63). Fortunately, our study revealed increases in the numbers of splenic CD4^+^ and CD8^+^ effector memory T cells, memory B cells, and activated B cells (Fig. 6), indicating the successful establishment of immunological memory. The above results suggest that RHΔ*Tg*pCaBP vaccination could establish a comprehensive immune memory program that supports rapid recall responses upon secondary challenge, which may constitute the core mechanism underlying durable protective immunity. However, additional cellular functional assays are still required to verify this speculative regulatory mechanism.

RHΔ*Tg*pCaBP showed favorable safety and efficacy in our acute challenge model, and several strategic optimizations are envisioned to increase its potential for clinical or veterinary application. In the present study, sulfadiazine was supplemented via free drinking water at the initial immunization procedure for three consecutive days. Sulfadiazine exerts anti-*Toxoplasma* activity by interfering with parasite pyrimidine biosynthesis and restraining tachyzoite proliferation (34, 35). Our *in vitro* observation results showed that sulfadiazine treatment reduces the parasite burden of both wild-type RH and RHΔ*Tg*pCaBP strains (Fig. S5), however, at the drug dosage adopted for mouse immunization, complete clearance of neither strain can be achieved *in vivo* (Fig. S6; Fig. 4D). Specifically, we harvested peritoneal fluid from mice infected with RH strain or RHΔ*Tg*pCaBP strain combined with sulfadiazine at 6 days post-infection and quantified tachyzoites via microscopic examination: viable tachyzoites were readily detectable in the RH + sulfadiazine group (Fig. S6A). Whereas no tachyzoites were observed in the RHΔ*Tg*pCaBP + sulfadiazine group (Fig. S6B), notably, mice receiving RHΔ*Tg*pCaBP + sulfadiazine exhibited positive *Toxoplasma*-specific IgG antibody responses (Fig. 4D). Thus, we hypothesize that during co-immunization, sulfadiazine alleviates acute tissue pathological lesions by limiting rapid tachyzoite replication, while residual RHΔ*Tg*pCaBP tachyzoites retain sufficient antigenicity to trigger protective adaptive immune responses.

Sulfadiazine, a sulfonamide-class antibiotic, faces stringent global usage regulations in animal husbandry. Although sulfadiazine was only applied as an experimental auxiliary agent only during the initial immunization procedure (400 mg/L via free drinking water for 3 days) in the present study, our research procedure still needs to be further optimized. The follow-up research will focus on developing antibiotic-free auxiliary immunization strategies. First, search for non-antibiotic natural small-molecule inhibitors (64) or herbal medicine substitutes to replace sulfadiazine. A study has revealed that curcumin was binded strongly to the active site of the DHFR protein (65). *In vitro* enzymatic affinity assays and MTT cytotoxicity tests verified that curcumin exerts direct inhibitory activity against *T. gondii* DHFR, with low host cell cytotoxicity at effective anti-parasitic concentrations (65). Another study validated curcumin and nano-curcumin as effective non-antibiotic immunomodulatory adjuvants that significantly elevate protective Th1-type cellular immunity and enhance antigen-specific IgG/IgG2a humoral responses, prolonging animal survival upon lethal parasite challenge without introducing antibiotic-related risks (66). This class of natural plant-derived compounds or natural small-molecule inhibitors represents a promising antibiotic-free substitute for sulfadiazine in future live attenuated vaccination protocols. Second, on the basis of our finding that pyrimidine metabolism is compromised in this mutant, a logical next step is to construct a double-knockout strain targeting both *Tg*p*CaBP* and a key gene in the pyrimidine metabolism pathway. Such a “dual-attenuation” strategy could further genetically cripple the parasite’s replicative capacity, thereby increasing safety, as seen in other attenuated live vaccine platforms, such as *T. gondii* mutants lacking both lactate dehydrogenases LDH1 and LDH2 (18). Furthermore, a controlled release delivery system (67) could be designed to encapsulate RHΔ*Tg*pCaBP tachyzoites with a slow-release pyrimidine metabolism pathway inhibitor within biodegradable nanoparticles. This approach would enable localized and sustained drug release to moderately restrain parasite proliferation *in vivo* over an extended period while simultaneously providing sufficient antigenic stimulation to elicit robust protective immunity. Although further optimization is needed, our study provides a foundational framework for future vaccine development.

## MATERIALS AND METHODS

### Parasite strains, animals, and ethics statement

The WT RH strain of *T. gondii* and the RHΔ*Tg*pCaBP knockout strain (23) were used in this study. Tachyzoites were maintained *in vitro* by serial passaging in monkey kidney adherent epithelial (Vero) cells in Dulbecco’s modified minimal Eagle’s medium (DMEM; Servicebio; Catalog No. G4515) supplemented with 2% fetal bovine serum (FBS; Excell Bio; Catalog No. FND500) and 100 µg/mL streptomycin (Beyotime; Catalog No. C0224) at 37°C in a 5% CO_2_ incubator.

Six-to-eight-week-old specific pathogen-free (SPF) female C57BL/6 mice were purchased from Liaoning Changsheng Biological Technology Company (Liaoning, China) and maintained under standard laboratory conditions with a 12-h light/dark cycle. All animal experiments were conducted in strict accordance with the guidelines of the Institutional Animal Care and Use Committee (IACUC) of Shenyang Agricultural University (permit no. SYXK <Liao > 2021-0010).

### Proteomic and bioinformatic analyses

For quantitative proteomic analysis, RHΔ*Tg*pCaBP and WT RH tachyzoites were harvested. Afterward, Percoll (GE Healthcare; Catalog No. 17-0891-09) gradient centrifugation was used to separate and purify the tachyzoites as previously described (68). Purified tachyzoites were washed with 1× cold phosphate-buffered saline (PBS) and sonicated three times in lysis buffer containing 8 M urea in the presence of 1% protease inhibitors (MCE; Catalog No. HY-K0010) in a sonicator (Scientz Ningbo; Catalog No. 01A566). The lysates were subsequently centrifuged at 12,000 × *g* (4°C) for 12 min to remove insoluble cell fragments, after which the samples were subjected to liquid chromatography‒tandem mass spectrometry (LC‒MS/MS) analysis at Jingjie PTM Biolab (Hangzhou) Co., Inc.

The data were processed using Spectronaut (v.18) software. Tandem mass spectra were searched against the *T. gondii* ME49 database (downloaded from ToxoDB) concatenated with the reverse decoy database. The false discovery rates (FDRs) of proteins, peptides, and PSMs were adjusted to <1%. Proteins with a log2 fold change value >0.5 or <−0.5 with and a *P*-value < 0.05 after Benjamini–Hochberg correction were further analyzed. For functional enrichment analysis of the differentially expressed proteins (DEPs), WolF PSORT software, which can be used to predict the subcellular localization of eukaryotic sequences, was used to predict the subcellular localization of DEPs; the KEGG online service tool KAAS was used to annotate the DEPs to their corresponding pathways using KEGG Mapper.

### Quantitative PCR

Total RNA was extracted from RHΔ*Tg*pCaBP and WT RH tachyzoites and reverse transcribed into cDNA. Quantitative PCR (qPCR) was performed using MonAmp ChemoHS qPCR Mix (Monad) on a QuantStudio 3 system (Thermo Fisher Scientific; Massachusetts; USA). The fold change was used to calculate the relative expression levels of the parasite genes employing the 2^−ΔΔCT^ method (69). The primers used are listed in Table S1. *T. gondii β-tubulin* was used as a housekeeping gene to determine the level of transcription of each target gene.

### *In vitro* parasite proliferation assays

The *in vitro* proliferation capabilities of RHΔ*Tg*pCaBP and wild-type (WT) *T. gondii* tachyzoites were assessed by flow cytometry using CFDA SE (Beyotime Biotechnology; Catalog No. C0051) and PI (Beyotime Biotechnology; Catalog No. C1052), respectively. Briefly, when CFDA SE was used, the purified tachyzoites were resuspended at 1× 10⁷/mL and labeled with an equal volume of 2× CFDA SE working solution (diluted from 200× stock) for 10–15 min at 37°C. Unbound dye was quenched by adding 10 mL of complete cell culture medium, followed by centrifugation, one wash, and an additional 5 min of incubation in complete medium at 37°C for label stabilization. For infection, Vero cells were seeded in 12-well plates at 3.5 × 10⁵ cells/well and grown to 80% confluence. CFDA SE-labeled RHΔ*Tg*pCaBP or WT tachyzoites were used to infect Vero cells at a multiplicity of infection (MOI) of 5:1. After 24 h of incubation to allow invasion, nonadherent parasites were removed by washing with serum-free DMEM, and the cells were cultured in fresh medium containing 1% FBS. Afterward, the cells were digested with trypsin-EDTA and repeatedly drawn with a 5-mL curved needle syringe. The suspension was then passed through 5.0-μm nucleopore filters to generate single-parasite tachyzoites, which were analyzed via flow cytometry (BD Aria III; BD Biosciences; New Jersey; USA). For PI staining, the entire cell culture process, including Vero cell seeding, tachyzoite infection, and 24-h incubation, was identical to that described for the CFDA SE labeling assay. The only difference was that PI staining was performed after the cell culture was completed and single-cell suspensions were obtained in strict accordance with the manufacturer’s instructions (Beyotime Biotechnology; Catalog No. C1052). The data were analyzed with FlowJo_v10 software to quantify parasite proliferation.

### Western blotting

Total proteins from *T. gondii* RHΔ*Tg*pCaBP and WT tachyzoites were obtained, as previously described (70), separated by 10% sodium dodecyl sulfate‒polyacrylamide gel electrophoresis (SDS‒PAGE) and transferred to polyvinylidene fluoride (PVDF) membranes (Bio-Rad; Catalog No. 162-0177). The membranes were blocked with 5% BSA and incubated with specific antibodies (Table S2) or mouse serum (1:1,000 dilution) from the unimmunized and immunized groups, followed by incubation with an HRP-conjugated secondary antibody (Table S2). The protein bands were visualized using an enhanced chemiluminescence (ECL) detection kit (Thermo Fisher Scientific; Catalog No. 32,106). The membranes were scanned using the Azure c300 system (Azure Biosystems, Dublin), and the signals were quantified using ImageJ software. The levels of the target proteins were normalized to those of β-tubulin (23).

### Growth and invasion assay

Briefly, 200 freshly egressed parasites were collected to infect Vero cells monolayers in six-well plates and incubated for 5–7 days. After three times washes with PBS, cells were fixed with 4% paraformaldehyde for 20 min and stained with crystal violet, as reported previously (23).

### *In vivo* virulence evaluation and sulfadiazine treatment

To evaluate the virulence of RHΔ*Tg*pCaBP, mice were intraperitoneally (i.p.) injected with 100, 500, 1,000, or 5,000 RHΔ*Tg*pCaBP or WT tachyzoites (*n* = 10 per group). At 48 h post-infection, the mice were provided drinking water containing 400 mg/L sulfadiazine (Sigma‒Aldrich; Catalog No. PHR1343) for three consecutive days, after which regular water was provided. Mouse survival was monitored daily for 80 days post-infection.

### Immunization schedule, vaccine safety, and protective efficacy assessment

To systematically evaluate the safety and protective efficacy of the RHΔ*Tg*pCaBP strain as an attenuated live vaccine, four experimental groups of C57BL/6 female mice were established: (i) Immunization Group 1 (*n* = 20): intraperitoneal injection of 100 RHΔ*Tg*pCaBP tachyzoites (0.2 mL/mouse); (ii) Immunization Group 2 (*n* = 20): intraperitoneal injection of 500 RHΔTgpCaBP tachyzoites (0.2 mL/mouse); (iii) WT RH control Group 1 (*n* = 20): intraperitoneal injection of 100 WT RH tachyzoites (0.2 mL/mouse); and (iv) WT RH control Group 2 (*n* = 20): intraperitoneal injection of 500 WT RH tachyzoites (0.2 mL/mouse). The mice were i.p. immunized three times at 21-day intervals. Sulfadiazine (400 mg/L) was administered via the drinking water for 3 days only starting at 48 h after the first immunization to further ensure safety. Seven days after the third immunization, 10 mice were sacrificed, and serum, lung, liver, spleen, and brain samples were collected. Serum samples were used to detect anti-Toxo IgG using an ELISA kit (MLBio, Catalog No. MM-45450M1) in strict accordance with the instructions, and the major organs (liver, lung, brain, and spleen) were obtained for safety assessment. Furthermore, the remaining 10 mice were challenged i.p. with 100, 500, or 1,000 WT RH tachyzoites, and survival was monitored daily for 30 days post-challenge.

### Histopathological examination

The major organs (liver, lung, brain, and spleen) from immunized and control mice were fixed in 4% paraformaldehyde for 24 h, embedded in paraffin, sectioned to a thickness of 5 μm, and stained with hematoxylin and eosin (HE). Slides were observed under a light microscope (Leica; Wetzlar, Germany) for detection of pathological lesions.

### Flow cytometry analysis of effective T cells

To explore the underlying mechanism of the protective efficacy induced by RHΔ*Tg*pCaBP immunization, the effector functions of T cells in peripheral blood were evaluated. Seven days post-challenge, peripheral blood cells were obtained. Then, red blood cells were lysed with red blood cell lysis solution (155 mM NH_4_Cl, 10 mM KHCO_3_, 0.11 mM EDTA Na_2_, pH 7.2). Furthermore, the cells were stained with a LIVE/DEAD Fixable Aqua Dead Cell Stain Kit (cat. no. L34966; Thermo Fisher Scientific) to discriminate between live and dead cells. To reduce nonspecific binding, the cells were preincubated with a purified anti-mouse CD16/32 antibody (cat. no. 101,310; BioLegend) to block Fc receptors. Cells were then stained with specific fluorescently labeled antibodies or corresponding isotype controls according to the manufacturers’ instructions; detailed antibody information is listed in Table S2. Flow cytometry was performed on a FACSAria III flow cytometer (BD Biosciences, San Jose, CA, USA). Cell populations with positive staining were identified using isotype controls or fluorescence-minus-one (FMO) controls, and the data were analyzed using FlowJo software v10.

### Flow cytometry analysis of memory T cells and memory B Cells

Further, we evaluated the formation of immune memory cells in the spleens of mice, after immunization and subsequent parasite challenge. Thirty days post-challenge, the mice were sacrificed, and the spleens were harvested and weighed. The spleen index was calculated to assess changes in splenic enlargement using the formula: spleen index = [spleen weight (mg) × 10/body weight (g)] ×100%.

Then, the spleens were mechanically dissociated, and single-cell suspensions were prepared by passing the samples through a 70-μm cell strainer (Beyotime Biotechnology; Catalog No. FSTR072). Single immune cells were obtained and analyzed as described in the “Methods” section of 2.9, and detailed antibody information is listed in Table S2.

### Statistical analysis

All the data are presented as the mean ± standard error of the mean (SEM). Statistical comparisons between two groups were made using unpaired two-tailed Student’s *t*-tests. *P*-values < 0.05 were considered to indicate statistical significance. All the statistical analyses were conducted using GraphPad Prism software (v9.0).

## Data Availability

The data supporting the findings of this article are provided within the manuscript and supplemental materials.

## SUPPLEMENTAL MATERIAL

File S1 spectrum.01827-26-s0001.docx Full uncropped gels and blots images.

Supplemental figures spectrum.01827-26-s0002.docx Fig. S1 to S6.

Table S1 spectrum.01827-26-s0003.xlsx Primers used in Fig. 1C and D.

Table S2 spectrum.01827-26-s0004.xlsx Specific antibodies used in this study.

Table S3 spectrum.01827-26-s0005.xlsx Differentially expressed proteins in Fig. 3A (RHΔTgpCaBP group vs WT group).

ASM does not own the copyrights to Supplemental Material that may be linked to, or accessed through, an article. The authors have granted ASM a non-exclusive, world-wide license to publish the Supplemental Material files. Please contact the corresponding author directly for reuse.
