# Supplementary material for: Role of Toxoplasma gondii calcium-binding protein (TgpCaBP) and its implications for the development of attenuated live vaccines
Source: Microbiol Spectr. 2026 Sep 17;14(10):e01827-26. doi: 10.1128/spectrum.01827-26 (PMC13689411; doi:10.1128/spectrum.01827-26)
Supplement: Supplementary file 1 [file spectrum.01827-26-s0001.docx]

**Full uncropped Gels and Blots images**

File 1. full uncropped Gels and Blots image in Figure 3C

File 2. full uncropped Gels and Blots image in Figure 4E
