# Supplementary material for: Role of Toxoplasma gondii calcium-binding protein (TgpCaBP) and its implications for the development of attenuated live vaccines
Source: Microbiol Spectr. 2026 Sep 17;14(10):e01827-26. doi: 10.1128/spectrum.01827-26 (PMC13689411; doi:10.1128/spectrum.01827-26)
Supplement: Supplementary file 2 [file spectrum.01827-26-s0002.docx]

**Supplementary Material**

**Role of *Toxoplasma gondii* calcium-binding protein (*Tg*pCaBP) and its implications for the development of attenuated live vaccines**

Yiwei Zhang^†^, Hongyu Li^†^, Yuqi Jing, Weisong Sun, Bin Wei, Zhiming Wei, Ning Jiang*

**Affiliations**:

Key Laboratory of Livestock Infectious Diseases, Ministry of Education, and Key Laboratory of Ruminant Infectious Disease Prevention and Control (East), Ministry of Agriculture and Rural Affairs, College of Animal Science and Veterinary Medicine, Shenyang Agricultural University, 120 Dongling Road, Shenyang 110866, China

^†^These authors contributed equally to this work.

* Corresponding author. Email: jiangning@syau.edu.cn

Telephone and Fax numbers: +86 15802458149 and +86 (0)24-88487156

**Supplementary material**

**Figure S1. Proteomic data quality control.** (A) Principal component analysis (PCA) was performed on the basis of the relative quantitative values of all the samples, and a PCA plot was generated to visualize the results. The horizontal and vertical axes display the variances explained by PC1 and PC2, respectively, with higher values indicating greater explanatory power. Intragroup clustering indicated the good repeatability of the replicate samples. (B) The inherent differences in the number of expressed proteins and their abundance distribution across samples and the number of proteins expressed within different intensity intervals were calculated and visualized as a stacked bar chart. The x-axis presents the sample names, the y-axis shows the percentage of proteins per sample, and distinct colors in the chart correspond to different ranges of log₁₀-transformed intensity values.

**Figure S2. Heatmap of the top 100 differentially expressed proteins between the RHΔ*Tg*pCaBP group and the WT group.** A heatmap was constructed using the MetWare Cloud, a free online platform for data analysis (https://cloud.metware.cn). Protein expression data were subjected to z score normalization; hypothetical proteins were excluded.

**Fig. S3. Histopathological analysis of mouse brain tissue slices.** Several mice immunized with 500 RHΔ*Tg*pCaBP tachyzoites exhibited inflammatory cell infiltration (black arrow), tissue damage and vacuolation (red arrow) in their brain tissue. Bar = 100 μm.

**Figure S4****. Flow cytometry** **gating strategy to analyze B-cell subsets.**  B lymphocytes (CD3^−^ CD19^+^), transitional B cells (CD3^−^ CD19^+^ CD45RA^+^ CD38^high^), follicular naïve B cells (CD3^−^CD19^+^ CD45RA^+^ CD38^low^), marginal zone B cells (CD3^−^CD19^+^ CD45RA^+^ CD38lowCD73^−^), follicular B cells (FoB cells; CD3^−^CD19^+^ CD45RA^+^ CD38^low^CD73^+^), activated B cells (CD3^−^CD19^+^ CD45RA^+^ CD38^low^CD80^+^), GC B cells (GC Bs; CD3^−^CD19^+^ CD45RA^−^CD38^low^), and memory B cells (B_memory_s; CD3^−^CD19^+^ CD45RA^−^ CD38^low^CD73^+^).

Figure S5. Sulfadiazine treatment reduces the parasite burden of both wild-type RH and RHΔ*Tg*pCaBP strains *in vitro*. Vero cells were infected with tachyzoites of wild-type RH or RHΔ*Tg*pCaBP strains and cultured with serial concentrations of sulfadiazine (0, 25, 50, and 100 μg/mL; CON = untreated control).

Figure S6. Microscopic images of peritoneal tachyzoites collected 6 days after different *T. gondii* strain infection combined with sulfadiazine treatment. Red arrows mark some of the viable *T. gondii* tachyzoites. Tachyzoites were observed in the RH + sulfadiazine group (A), while no intact tachyzoites were observed in the RHΔTgpCaBP + sulfadiazine group (B). Scale bar ＝ 50 μm.
